# Prevalence of sexualized drug use and risk of HIV among sexually active MSM in East and South Asian countries: systematic review and meta‐analysis

**DOI:** 10.1002/jia2.26054

**Published:** 2023-01-04

**Authors:** Laura Nevendorff, Sophia E. Schroeder, Alisa Pedrana, Adam Bourne, Mark Stoové

**Affiliations:** ^1^ Disease Elimination Program Burnet Institute Melbourne Victoria Australia; ^2^ School of Public Health and Preventive Medicine Monash University Melbourne Victoria Australia; ^3^ HIV AIDS Research Center, Atma Jaya Catholic University of Indonesia South Jakarta Indonesia; ^4^ Australian Research Centre in Sex, Health and Society La Trobe University Melbourne Victoria Australia; ^5^ Kirby Institute University of New South Wales Sydney New South Wales Australia

**Keywords:** sexualized drug use, men who have sex with men, East‐South Asia, sexual risk behaviours, systematic review, meta‐analysis

## Abstract

**Introduction:**

Sexualized drug use (SDU), the use of psychoactive drugs in the context of sexual intercourse, has been identified as a risk factor for HIV among men who have sex with men (MSM) in Asia. Given the distinct social and cultural context of same‐sex relationships and drug‐using practice in Asia, we aimed to describe the prevalence of SDU in East and South Asian countries and its associations with condomless anal sex (CAI) and HIV status. Synthesizing SDU research in this region, including SDU definitions, prevalence and outcomes, provides insights to inform future research and improved programme planning, resourcing and advocacy.

**Methods:**

We systematically searched OVID Medline, OVID EMBASE, OVID Global Health, CINAHL, PsycINFO and SCOPUS publication databases for scientific articles published from 1990 to 2022 measuring SDU among MSM in East and South Asian countries. A narrative synthesis was utilized to describe key study attributes and findings, and meta‐analyses using random pooled effect models were used to estimate SDU prevalence and its associations with CAI and HIV status. Subgroup meta‐analyses, sensitivity analysis and assessment of publication bias examined potential sources of heterogeneity for the pooled SDU prevalence estimates.

**Results and discussion:**

Of the 1788 publications screened, 49 publications met the selection criteria and 18 were suitable for meta‐analyses. Findings highlight SDU definitions distinct from other regions but inconsistencies in the definition of SDU between studies that have been highlighted in research elsewhere. The pooled prevalence of recent SDU (past 12 months) was 13% (95% CI = 10–16%; *I* ^2^ = 97.6) but higher when studies utilized self‐administered surveys (15%; 95% CI = 12–19%; *p*<0.05). SDU was associated with greater odds of CAI (pooled odds ratio [OR] = 3.21; 95% CI = 1.82–5.66) and living with diagnosed HIV (OR = 4.73; 95% CI = 2.27–8.21).

**Conclusions:**

SDU is common among MSM in East and South Asian countries, but varying SDU definitions limit between‐study comparisons. Responses to SDU‐related harms should consider local contexts, including specific drug types used and their relative risks.

## 1 INTRODUCTION

The United Nations Office on Drugs and Crimes has recently emphasized the urgency of addressing intersecting drug use and sexual practices by recommending targeted interventions for HIV prevention, particularly among men who have sex with men (MSM) [1]. The need for effective responses is driven by mounting evidence that sexualized drug use (SDU) is often associated with behaviours that present a greater risk for HIV and other sexually transmitted infection (STI) transmission [2, 3]. SDU is typically characterized as the use of psychoactive substances (e.g. methamphetamine, mephedrone and GHB‐gamma hydroxybutyrate) before or during sexual activity [4, 5]. Such behaviours are typically associated with condomless anal intercourse (CAI) between serodiscordant partners in the absence of biomedical prevention technologies, group sex, higher numbers of sex partners and the sharing of injecting equipment [6, 7]. The reported prevalence of SDU among MSM varies by geography and recall period. According to the most recent European MSM Internet Survey, SDU was practised in the previous 12 months by 18% of MSM surveyed [8]. SDU practice among MSM in Latin American countries reached 24% in the previous year [9], but Brazil recorded 36% MSM practised SDU in the past 3 months [10]. SDU has been reported as more common among groups of MSM engaging in HIV and other STIs high‐risk behaviours, including MSM living with HIV, male sex workers and young MSM [11, 12, 13, 14, 15]. While there is also evidence to suggest the use of HIV pre‐exposure prophylaxis (PEP) may be more common among MSM who practice SDU [16, 17, 18], pre‐exposure prophylaxis (PrEP) programme coverage remains limited in many countries [19, 20].

Concerns regarding the use of drugs in sexual contexts among MSM have also emerged in Asia, where the practice has been described in various countries, including China, Indonesia, Malaysia and Thailand [21, 22, 23, 24]. The types of drugs being used in sexual contexts in Asia are varied and include stimulant drugs (e.g. methamphetamine and amphetamine), amyl nitrate/alkyl nitrate—colloquially known as *poppers*, hallucinogenic drugs (e.g. 5‐methoxy‐N or *foxy‐5*), erectile dysfunctional drugs and/or opiate‐based drugs (e.g. Tramadol). The types of drugs identified as forming part of the SDU environment in Asia are somewhat divergent from other regions, particularly in Europe where SDU has been traditionally framed around the term *chemsex* and defined almost exclusively around the use of mephedrone, crystal methamphetamine and GHB/GBL [25]. The divergent characterization of SDU in Asia is likely due to the relative cost and availability of drug types, which is influenced by local manufacturers and drug distribution chains [26, 27].

Ongoing high rates of HIV diagnoses among key populations in Asia remain a concern, including among MSM [28], where criminalization of same‐sex relationships, cultural norms and stigma impede effective responses [29]. In light of the distinctive nature of SDU in Asia and its putative role in contributing to HIV transmission risk, understanding the regional prevalence and patterns of SDU is a key step in responding [30], and can assist in informing programme resourcing, advocacy and planning [31]. Previous systematic reviews of SDU practice among MSM have all conducted qualitative syntheses, with heterogeneous methodological and contextual factors limiting SDU prevalence estimations [6, 32, 33]. While a recent review examined SDU and *chemsex* practice in Asia, it did not focus specifically on MSM and included only qualitative studies [34].

Taking into account the regional context of HIV, sex between men and patterns of drug use in Asia, we conducted a systematic review and meta‐analysis to describe the prevalence of SDU in East and South Asian countries and its associations with CAI and HIV status.

## 2 METHODS

This review followed Preferred Reporting Items for Systematic Review and Meta‐Analysis (PRISMA) recommendations [35] and was registered with the International Prospective Register of Systematic Reviews (PROSPERO) (registration number: CRD42020197214).

### 2.1 Search strategy

Initial searches were conducted in multiple electronic databases to identify suitable keywords and develop an optimal search strategy in consultation with an expert librarian. On 26 September 2022, the final search of peer‐reviewed studies was conducted using six databases OVID Medline, OVID EMBASE, OVID Global Health, CINAHL, PsycINFO and SCOPUS (Appendix S1). We utilized multiple Medical Subject Heading search terms and synonyms across five topic area domains—MSM and other descriptions of sexuality and same‐sex relations between men; drug types, SDU and other terms used to describe drug use in sexual settings; PrEP and PEP; and list of East and South Asian countries. PrEP and PEP were included as domains because PrEP or PEP studies commonly assess SDU practice among MSM [6]. Database searches also included publications from conference proceedings to ensure the inclusion of more recent studies [36]. The search was restricted to studies conducted in East and/or South Asian countries as defined by the World Bank economic country classifications [37], with the exclusion of Pacific countries given the sexual practice and drug use contextual, political and cultural differences [38], and human studies published from 1990 to the search date, reflecting the period when drug use in sexual contexts among MSM emerged in the literature. We also reviewed reference lists of retrieved studies to check if the search missed relevant publications.

### 2.2 Eligibility, extraction, screening and measure

The results of all searches were entered into the Covidence systematic review software (Veritas Health Innovation, Melbourne, Australia) for the screening process. All duplicate records within and between different bibliographic databases were automatically identified and removed by the software before conducting the formal review process. Initially, two reviewers (LN and SS) independently screened article titles and abstracts to identify primary relevant studies for full‐text review, with discrepancies resolved through discussion.

The inclusion and exclusion criteria of this review accorded with the Condition, Context and Population mnemonic for reviews assessing prevalence data [39]. We included studies that reported the proportion of MSM who practiced SDU or provided sufficient data to calculate this proportion. To accommodate regional variations in commonly used recreational drugs in East and South Asia, we define SDU practice as the use of any type of drug in the context of sexual activity [26, 40]. Studies of SDU among broader populations were included if data were disaggregated by MSM status. Studies were excluded if they reported drug use not in a sexual context, exclusively reported SDU among cis‐women, heterosexual cis‐men or transgender people not identifying male‐to‐male sex practices; provided insufficient data to characterize and/or measure the prevalence of SDU; were based on Asian‐born men in non‐Asia settings; or were non‐English language articles. Full‐text reviews and data extractions were conducted by the first author. Where a study resulted in multiple publications from the same sample, the study that reported data that were the most up‐to‐date, comprehensive or relevant to the study aim was included. Included studies were uploaded into Mendeley (version 1.19, Mendeley Ltd.) for data extraction purposes.

Data were extracted into a purpose‐built Excel spreadsheet. Data fields included: author, year of publication, the country where research was conducted, data collection period, study population, the definition of SDU, recall period for SDU, study design (sampling procedure and data collection method), total participants, participants reporting SDU, selected socio‐demographics of study participants, odds ratio (OR) and 95% confidence intervals (95% CI) for CAI, HIV status and/or other variables significantly associated with SDU. In prospective cohort studies, only baseline data were extracted. In cases where a study reported serial surveillance surveys, data from the most recent year were extracted. For the SDU pooled prevalence estimate, where studies recruited and reported disaggregated data from key populations of HIV (e.g. MSM sex workers, MSM who inject a drug and transgender people) or multiple countries, only SDU data from general MSM or country in East or South Asian regions were extracted.

### 2.3 Data synthesis and analysis

A narrative synthesis was undertaken to characterize included studies by key attributes, including the measurement and prevalence of SDU and sexual practices associated with SDU.

We restricted the meta‐analysis to enhance homogeneity in the research design to avoid biased estimates [41]. First, to ensure comparability of the SDU prevalence outcome [42], we only included studies that measured SDU within the past 12 months and greater than the past month (i.e. past 3 months/3PM, past 6 months/6PM or past 12 months/12PM), in addition to the general inclusion and exclusion criteria for the systematic review described above. Second, included only the majority of studies that described polydrug SDU as opposed to single drug use in SDU (poppers only = 3, methamphetamine only = 2). Third, we restricted meta‐analysis to the general MSM population and we also excluded studies that reported SDU prevalence in the context of specific partner types, behaviours or sub‐populations (e.g. those focusing on SDU among MSM with regular partners only, SDU with internal ejaculation only and MSM sex workers) that could bias general prevalence estimates [33]. SDU prevalence for each study was computed using standard error and a 95% confidence interval to derive a pooled prevalence estimate using the Clopper–Pearson (exact) method [43]. The quality of the final studies included in the meta‐analysis was appraised using a critical appraisal checklist for studies reporting prevalence data from the Joanna Briggs Institute [39] (Appendix S2).

Meta‐analyses of the association between SDU and CAI and HIV‐positive status were conducted using data from studies that reported OR or provided sufficient information for the manual calculation of ORs for SDU among MSM who did and did not report CAI and among MSM with or without diagnosed HIV (self‐report or clinically confirmed). ORs and 95% confidence intervals were first transformed to the logarithmic scale. The random effect empirical Bayes model, which takes account of within‐ and between‐study variance, was used to measure the combined effect size for SDU prevalence, CAI and HIV infection outcomes [44, 45].

Heterogeneity between studies was assessed with *Q*‐statistics (*p* <0.05 was considered indicative of statistically significant heterogeneity, and the *I* ^2^ statistics >75% were considered high heterogeneity) [46]. Potential sources of heterogeneity for SDU prevalence were explored through subgroup meta‐analyses using a random‐effects model. We examined pooled SDU prevalence estimates [47] by age groups (below and above 30 years old); recall period (past 3, 6 and 12 months); economic level (high income, middle income and low income [37]); sampling methods (probability and non‐probability); recruitment methods (offline, online/mixed online and offline); data collection methods (interviewer‐administered questionnaire, self‐administered/computer/telephone‐assisted interview); the number of geographical locations (single or multiple locations); and study quality (low or high quality). Tests for subgroup differences in CAI and HIV status were not conducted due to the limited number of studies [48].

Sensitivity analyses were employed to examine the consistency of the pooled estimates by excluding any individual study from the meta‐analysis [49]. Publication bias for SDU prevalence was assessed by visual inspection of the funnel plot and further statistically confirmed through Begg's test, which is appropriate for non‐binary outcomes and large study samples (>14) [50, 51].

All statistical analyses and graphical representations were carried out within STATA (SE V.17.0, StataCorp LLC, Texas). Microsoft Excel (V.16.52) was utilized to create an additional graph to assess sensitivity analysis.

## 3 RESULTS AND DISCUSSION

**Figure 1:**
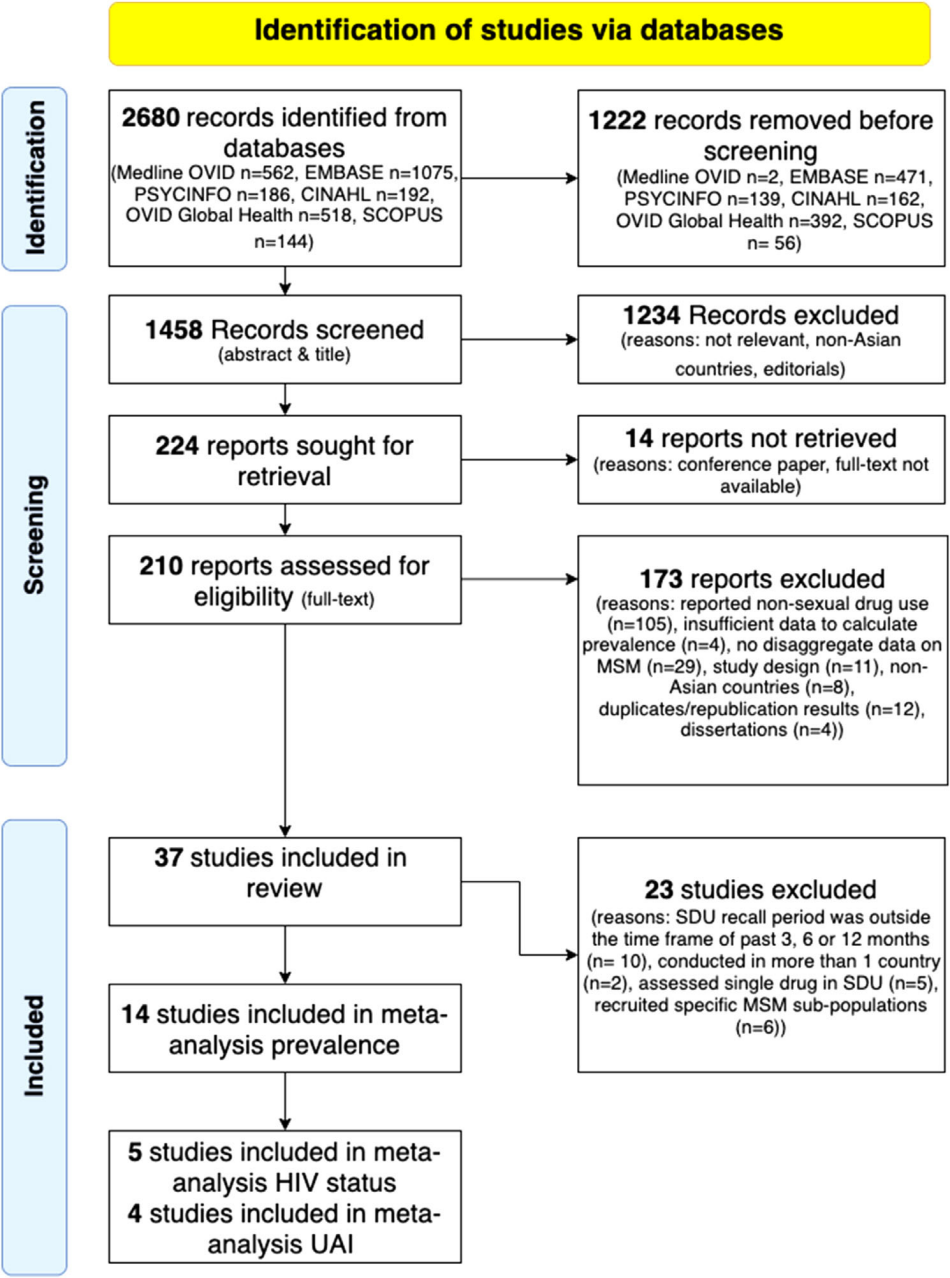
PRISMA flow diagram. This depicts a PRISMA diagram that details our search and selection process applied during the review.

We identified 3362 records, of which 1574 were removed as duplicate records. In total, 1788 titles and abstracts were screened, and 1488 studies were excluded because they did not measure or report SDU prevalence, were not conducted in East and/or South Asian countries or were editorials. Of the remaining 300 records, full‐text descriptions were available for 271 articles. From this, 222 records were excluded on the basis of only reporting drug use not in the context of sex (*n* = 138), insufficient data to calculate SDU prevalence (*n* = 7), disaggregated data on MSM not reported (*n* = 34), inappropriate study design (e.g. case report, qualitative studies, systematic review and case study) (*n* = 15), disaggregated data on East and/or South Asian countries not available (*n* = 8), duplication or republication of results (*n* = 14) or dissertations (*n* = 6). After exclusions, 49 studies were included in the qualitative synthesis. After applying exclusions based on the SDU recall period case definition, 18 studies were included in the meta‐analysis of SDU prevalence. After applying further restrictions on the basis of prevalence data and predictors of being disaggregated by CAI and HIV status, four studies were included in the meta‐analysis of SDU and CAI, and five in the meta‐analysis of SDU and diagnosed HIV. Study inclusions and exclusions are shown in Figure 1.

### 3.1 Characteristics of the included studies

The 49 included studies [16, 21, 52, 53, 54, 55, 60, 61, 62, 63, 64, 65, 66, 67, 68, 69, 70, 71, 72, 73, 74, 75, 76, 77, 78, 79, 80, 81, 82, 83, 84, 85, 86, 87, 88, 89, 90, 91, 92, 93, 94, 95, 96, 97, 98, 99] were published between 2009 and 2022 and recruited 46,157 MSM participants from 10 East and/or South Asian countries. Studies were conducted in China (*n* = 20), Hong Kong (*n* = 10), Thailand (*n* = 6), Malaysia (*n* = 4), multiple Asian countries (*n* = 2), Cambodia (*n* = 1), Indonesia (*n* = 1), Japan (*n* = 1), Taiwan (*n* = 1), Singapore (*n* = 1) and Vietnam (*n* = 1). The mean age of study participants was 29 years (mean age range = 16.4–37.5). Most studies recruited from non‐specific general MSM populations (*n* = 36); of these, four studies excluded participants with diagnosed HIV. The remaining studies described SDU in specific MSM populations: MSM diagnosed with HIV (*n* = 5), MSM aged 25 or younger (*n* = 5), MSM sex workers (*n* = 2) and MSM using dating apps (*n* = 1) (Table 1).

**Table 1:** Characteristics of included studies for qualitative and quantitative synthesis

									**UAI**	**HIV**	
**No**	**Authors/year of publication**	**Country of origin**	**Data collection period**	**Study population**	**Definition/category**	**Recall period**	**Methods (study design, source of study population, sampling technique and method of measurement**)	**# SDU event /total (%)**	**(OR; 95% CI)/time frame**	**(OR; 95% CI)**	**Other significant factor(s) associated with SDU (OR/aOR, 95% CI, *p*‐value)**
1	Kwan et al. (2022) [98]	Hong Kong/high income	March 2019–Nov 2020	MSM HIV positive	Use of psychotropic drugs for sex/SDU—general type	Past 12 months	Cross‐sectional, hospital; convenience; self‐administered questionnaire	152/356 (42.6%)	n.a	n.a	Willingness to participate in functional cure trial (OR 3.42, 1.25–9.33, *p*<0.05)
2	Zhang et al. (2022)^a^ [97]	China/upper‐middle income	August–Sep 2020	General MSM	Use of any of the following psychoactive substances before or during sexual intercourse: ketamine, methamphetamine, cocaine, cannabis, ecstasy, Dormicum/Halcion/Erimin5/non‐prescription hypnotic drugs, heroin, cough suppressant, gamma‐hydroxybutyric acid (GHB)/gamma‐butyrolactone (GBL), 5‐methocy‐N‐N‐diisopropyltryptamine (Foxy) and mephedrone/SDU‐specific type	(i) Lifetime and (ii) past 6 months	Cross‐sectional, online and community method, convenience, self‐administered questionnaire	Lifetime 45/595 (7.6%) Past 6 months 22/595 (3.7%)	n.a	n.a	Utilizing any HIV testing after the COVID‐19 outbreak (Lifetime SDU OR 3.15,1.53–6.50, *p*<0.005; aOR 2.94, 1.41–6.06, *p*<0.005)
3	Jiang et al. (2022) [96]	China/upper‐middle income	June 2017–April 2018	General MSM	Poppers use before sex over the last 6 months/poppers—specific single drug type	Past 6 months	Cross‐sectional, community, convenience, self‐administered questionnaire	167/500 (33.4%)	n.a	n.a	Multiple sex partners (OR 2.4, 1.6–3.6, *p*<0.001)
4	Eger et al. (2022)^a^ [88]	Malaysia/upper‐middle income	June–July 2020	General MSM	Use of recreational drugs (e.g. crystal methamphetamine, ketamine, ecstasy and gamma‐hydroxybutyrate G) before or during sex/chemsex‐specific type	Past 6 months	Cross‐sectional, online, convenience, audio computer self‐interview, self‐administered questionnaire	42/355 (11.8%)	n.a	n.a	Predicting PrEP use (OR 4.8, 2.4–9.5, *p*<0.005)
5	Fan et al. (2022) [95]	China/upper‐middle income	January–April 2019	Young MSM	Use any of the listed recreational drugs during sex: alkyl nitrites, crystal methamphetamine, ketamine/SDU‐specific type	Lifetime	Cross‐sectional, online and community method, convenience, self‐administered questionnaire	130/447 (29%)	n.a	n.a	Seeking sex partner via gay app (OR 1.52, 1.2–2, *p*<0.05)
6	Yang et al. (2022)^a^ [94]	China/upper‐middle income	August 2019–January 2020	General MSM	Substance use during anal sex/SDU general type	Past 6 months	Cross‐sectional; community; venue‐based sampling and peer referral; face‐to‐face interview	82/446 (18.3%)	n.a	n.a	n.a
7	Shrestha et al. (2022) [99]	Malaysia/upper‐middle income	July 2018–March 2020	General MSM	Any use of crystal methamphetamine, gamma‐hydroxubutyrate, gamma‐butryrolactone or 5‐methoxy‐N, N‐diisoprypyl tryptamine (foxy) before or during sexual activity/SDU‐specific type	Lifetime	Cross‐sectional, community, RDS, face‐to‐face interview	82/376 (21.8%)	n.a	n.a	n.a
8	Dong et al. (2022) [93]	China/upper‐middle income	n/a	General MSM	Use any drugs including ecstasy, crystal‐meth, marijuana, cocaine and others before sex/SDU‐specific type	Lifetime	Cross‐sectional, community, snowball, face‐to‐face interview	17/547 (3.1%)	n.a	n.a	Predictor for the risk of HIV (OR 4.5, 1.2–16.4, *p*<0.05)
9	Kongjareon et al. (2022) [92]	Thailand/upper‐middle income	n/a	Young MSM	Use drugs (crystal meth, ketamine and ecstasy) before or during sexual activities/SDU‐specific type	Lifetime	Cross‐sectional, community, convenience, self‐administered questionnaire	16/89 (17.9%)	n.a	n.a	Polydrug use (OR 9.14, 2.7–30.6, *p*<0.01)
10	Duan et al. (2021)^a^ [91]	China/upper‐middle income	Sep 2017–January 2018	General MSM	Sex after drugs use/SDU general type	Past 6 months	Cross‐sectional, community method, convenience, self‐administered questionnaire	87/652 (13.3%)	n.a	n.a	n.a
11	Tan et al. (2021) [90]	Singapore/high income	May–September 2019	Young MSM	Ever used amyl nitrites/poppers‐specific single type Ever used crystal methamphetamine/meth‐specific single type Ever used gamma‐dydroxybtyrate/gamma‐butyrolactone/GHB/GBL‐specific single type	Lifetime	Cross‐sectional, community method, convenience, self‐administered questionnaire	161/570 (28.3%) (poppers) 27/570 (4.7%) (meth) 27/570 (4.7%) (GHB/GBL)	n.a	n.a	n.a
12	Lee et al. (2021) [89]	Taiwan/high income	April 2017–July 2020	MSM HIV positive	Use recreational drugs listed (cannabis, cocaine, amphetamine, methamphetamine, ecstasy, GHB, ketamine, nimetazepam, flunitrazepam, heroin, 5‐metoxy‐diispropyltrptamine, mephedrone, lyseragic acid diethylamine and/or poppers) before or during sexual activities/SDU‐specific type	Past 1 year at HIV dignosis (period 1=2015–2016; period 2= 2017–2018; period 3= 2019–2020)	Cross‐sectional, hospital; convenience; self‐administered questionnaire	198/577 (34.3%) (Total)	n.a	n.a	n.a
13	Ng et al. (2020)^a^, ^b^ [52]	Malaysia/upper‐middle income	July 2017–February 2018	General MSM	Ever use of psychoactive substances (crystal meth/“Batu”/ice, ketamine, ecstasy, poppers and gamma hydroxybutyrate/gamma butyrolactone [GHB/GBL]) before or during anal intercourse/SDU—specific type	Lifetime	Cross‐sectional; online method; convenience; self‐administered questionnaire	140/622 (22.5%)	2.16 (1.45–3.23)/past 3 months	2.16 (1.45–3.23)	Significant suicidal risk (OR 1.84, 1.13–2.99, *p*<0.05)
14	Chen et al. (2020) [53]	China/upper‐middle income	July–October 2013	MSM HIV positive	Use of illicit drugs (e.g. heroin, meth, cocaine and popper/ RUSH) during anal intercourse in the past 30 days/SDU—specific type	Past 1 month	Cross‐sectional; community; convenience; face‐to‐face interview	34/415 (8.2%)	n.a	n.a	Life satisfaction (aOR 0.89, 0.83–0.96; *p*<0.01); one regular sex same‐sex partner (OR 0.27, 0.12–0.63, *p*<0.01); non‐regular same‐sex partners: 1 person (OR 7.20, 2.27–22.84, *p*<0.001); ≥ 2 people (7.56, 2.76–21.39, *p*<0.001)
15	Jiang et al. (2020) [57]	China/upper‐middle income	1 July 2016 and 30 June 2017	General MSM	Use of illegal drugs before condomless‐anal sex in the past 3 months/SDU—general type	Past 3 months	Cross‐sectional; community; snowball; face‐to‐face interview	8/975 (0.8%)	n.a	n.a	n.a
16	Wong et al. (2020)^a^, ^b^, ^c^ [55]	Hong Kong/high income	April and September 2017	General MSM	Use of recreational drugs (including poppers/rush, EDA, GHB, methamphetamine, ketamine, MDMA/ecstasy, cocaine, foxy/0 capsule and cannabis) before or during sex in the past 6 months/SDU—specific type	Past 6 months	Cross‐sectional; community and online method; convenience sample; self‐administered questionnaire	356/3043 (11.6%)	6.05 (4.3–8.51)/past 6 months	7.35 (5.3–10.19)	Age >29 (OR 1.49, 1.19–1.87, *p*<0.05); non‐Chinese (OR 2.22, 1.54–3.19, *p*<0.05); monthly income ≥ HKD 30,000 (1.44, 1.13–1.83, *p*<0.05); STI diagnosis 12PM (OR 5.74, 4.43–7.45, *p*<0.05); newly detected HIV infection (0.27, 0.12–0.64, *p*<0.05); sex with regular partner (2.7, 1.98–3.68, *p*<0.05); sex with non‐regular partners (2, 1.49–2.68, *p*<0.05); sex with male client (1.49, 1.1–2.03, *p*<0.05); sex with partner overseas (2.49, 1.96–3.16, *p*<0.05); >1 sex
											partner (OR 3.09, 1.98–4.83, *p*<0.05); group sex (7.19, 5.58–9.26, *p*<0.05); alcohol before sex (2.43, 1.9–3.1, *p*<0.05); tested for HIV 12PM (1.77, 1.3–2.3, *p*<0.05); heard of PrEP (3.52, 2.58–4.78, *p*<0.05); intend to take PrEP (2.78, 2.1–3.7, *p*<0.05); have taken PrEP (8.61, 5.64–13.14, *p*<0.05)
**17**	Wang et al. (2020)^a^, ^b^, ^c^ [56]	Hong Kong/high income	April 2018–July 2019	General MSM	Use of any of the following psychoactive substances before or during sexual intercourse: ketamine, methamphetamine, cocaine, cannabis, ecstasy, Dormicum/Halcion/Erimin 5/non‐prescription hypnotic drugs, heroin, cough suppressant (not for curing cough), amyl nitrite (popper), GHB/GBL, 5‐methoxy‐N, N‐dipropyltryptamine (Foxy) and mephedrone/SDU—specific type The use of ketamine, methamphetamine, cocaine, GHB/GBL or mephedrone before or during sexual intercourse/chemsex	Past 12 months	Prospective cohort; community and online method; venue‐based sampling, peer referral and online outreach; telephone interview	88/600 (14.6%) (SDU)^e^ 40/600 (6.6%) (chemsex)	4.37 (1.87–10.18)/past 12 months	6.85 (1.97–23.87)	Currently on PrEP (OR 8, 2.53–25.4, *p*<0.001); history of STI (OR 3.31, 1.5–7.3, *p*<0.01); sex with non‐regular sex partners (OR 20.2, 2.7–149.9, *p*<0.01)
**18**	Wei et al. (2020)^a^ [21]	China/upper‐middle income	April 2019 and June 2019	General MSM	Drug use during sex within the past 6 months/SDU—general type	Past 6 months	Cross‐sectional; community; convenience; self‐administered questionnaire	109/578 (18.9%)	n.a	n.a	Monitoring type of intimate partner violence (IPV) (OR 3.37, 1.2–6.44, *p*<0.001); controlling type of IPV (OR 2.5, 1.5–4.2, *p*<0.001); emotional type of IPV (OR 2.2, 1.3–3.6, *p*<0.01)
**19**	Jiang et al. (2020) [57]	China/upper‐middle income	May–November 2017	General MSM	Rush popper use prior to sex in the last 6 months/poppers—specific single drug type	Past 6 months	Cross‐sectional; HIV testing clinics; convenience; self‐administered electronic questionnaire	340/976 (43.84%)	n.s	1.85 (1.13–3.03)	Multiple sex partners (OR 2.4, 1.8–3.2, *p*<0.001); IPV (OR 1.9, 1.3–2.75, *p*<0.01); alcohol use (OR 1.9, 1.13–3.03, *p*<0.05)
**20**	Wang et al. (2020)^a^, ^b^ [16]	Hong Kong/high income	April–December 2018	General MSM	Use of any psychoactive substances (ketamine, methamphetamine, cocaine, cannabis, ecstasy, Dormicum/Halcion/Erimin 5/non‐prescription hypnotic drugs, heroin, cough suppressant (not for curing cough), amyl nitrite (popper), GHB/GBL (γ‐hydroxy‐butyrate), 5‐methoxy‐N, N‐diisopropyltryptamine (Foxy) and mephedrone before/during sexual intercourse in the past 12 months/SDU—specific type Use of some specific psychoactive substances (methamphetamine,mephedrone, γ‐hydroxybutyrate [GHB/GBL], ketamine and cocaine) before/during sexual intercourse/chemsex—specific type	Past 12 months	Cross‐sectional; community and online method; online outreach, peer referral, online advertisement; telephone interview	82/580 (14.1%) SDU 37/580 (6.4%) chemsex	2.47 (1.54–3.98)/past 12 months	n.a	Education college and above (OR 0.55, 0.31–0.98, *p*<0.05); part‐time employment/unemployed/retired (OR 1.97, 1.13–3.43, *p*<0.05); HIV service utilization (OR 1.9, 1.1–3.1, *p*<0.05); history of STI (OR 2.52, 1.51–4.21, *p*<0.0001), anal sex with non‐regular sex partners (OR 7.3, 3.4–15.4, *p*<0.001); multiple sex partners (OR 4.8, 2.3–10.2, *p*<0.001); on PrEP (7.6, 3.2–17.9, *p*<0.001)
**21**	Kwan et al. (2020) [58]	Hong Kong/high income	2016 and 2018	General MSM	Drug use in the context of facilitating sex/SDU—general type	Lifetime	Cross‐sectional; hospitals; convenience; self‐administered questionnaire	227/371 (61.1%)	n.a	n.a	Connected in the same sex‐networking (OR 1.8, 1.18–2.74, *p*<0.01)
**22**	Kwan and Lee (2019) [59]	Hong Kong/high income	August and September 2016	General MSM	The use of psychotropic drugs for sex/SDU—general type	Lifetime	Cross‐sectional; online; convenience; self‐administered questionnaire	51/453 (11.3%)	n.a	n.a	n.s
**23**	Yu et al. (2019) [60]	China/upper‐middle income	December 2014–June 2015	MSM sex workers	Use of substance during sex in the past 3 months/SDU—general type	Past 3 months	Cross‐sectional; community; convenience multi‐stage sampling; face‐to‐face interview	224/330 (67.9%)	n.a	n.a	n.a
**24**	Zhang et al. (2019)^a^, ^c^ [61]	China/upper‐middle income	May 2013–December 2017	General MSM	Drug use during anal intercourse in the past 6 months/SDU—general type	Past 6 months	Cross‐sectional; community; snowball; face‐to‐face interview	19/1611 (1.4%)	n.a	7.47 (2.95–19.15)^d^	n.a
**25**	Wang et al. (2018)^a^ [62]	China/upper‐middle income	March 2014–August 2014	General MSM	Drug use before sex during the 6 months prior to the study/SDU—general type	Past 6 months	Cross‐sectional; community; snowball; face‐to‐face interview	17/546 (3.1%)	n.a	n.a	n.a
**26**	Chard et al. (2018) [63]	Thailand/upper‐middle income	n/a	General MSM	The last time you had sex with [partner] were you high on drugs?; “sex,” “buzzed,” “drunk,” “high” and “drugs” were all self‐defined/SDU—general type	Last sex	Cross‐sectional; online; convenience; self‐administered questionnaire	5/238 (2.1%)	n.a	n.a	n.a
**27**	Tang et al. (2017)^a^ [64]	China/upper‐middle income	September 2014–October 2014	General MSM	Any sex while using recreational drugs (including, but not limited to poppers or rush [amyl nitrite^g^], ecstasy and crystal methamphetamine) in the last 12 months (yes or no)/SDU—specific type	Past 12 months	Cross‐sectional; online; convenience; self‐administered questionnaire	324/1424 (22.8%)	n.a	n.a	Sexual orientation disclosure to health professional (OR 0.66, 0.48–0.9, *p*<0.05)
**28**	Wang et al. (2017) [66]	China/upper‐middle income	April 2013–April 2014	General MSM	Self‐reported having ever used nitrate inhalant during male–male sex in the past 6 months/poppers—specific single drug type	Past 6 months	Prospective cohort; community and online methods; convenience; face‐to‐face interview	152/510 (29.8%)	n.s	2.0 (1.1–3.71)	Age 18–30 (OR 2.2, 1.4–3.3, *p*<0.01); education college/higher (OR 2.4, 1.6–3.5, *p*<0.01); income ≥700 USD (OR 2.6, 1.6–4.2, *p*<0.001); single marital status (OR 2.3, 1.4–3.8, *p*<0.01); monthly income >700 USD (OR 2.6 (1.6–4.2, *p*<0.001); drank alcohol (OR 2.3, 1.4–3.6, *p*<0.01); ≤ 10 lifetime sex partners (OR 3, 2.1–4.5, *p*<0.01); seeking sex partners through internet (OR 7.1, 3.2–15.6, *p*<0.01); ≥2 sex partners (OR 2.9, 1.9–4.3, *p*<0.001)
**29**	Vu et al. (2017) [67]	Vietnam/lower‐middle Income	September–December 2014	General MSM	Methamphetamine use before or during sex in the past 3 months/Meth—specific single drug type	Past 3 months	Cross‐sectional; community; convenience; face‐to‐face interview	89/622 (14.3%)	n.a	n.a	n.a Note: The study measured prevalence ratio.
**30**	Ren et al. (2017) [68]	China/upper‐middle income	14 May –17 May 2016	General MSM	Anal sex after drug use/SDU—general type	Lifetime	Cross‐sectional; online; convenience; self‐administered questionnaire	1852/5996 (30.9%)	n.a	n.a	n.a
**31**	Choi et al. (2017) [69]	Hong Kong/high income	n/a	Young MSM	(i) Had ever used drugs recreationally in conjunction with sexual intercourse; (ii) used drugs recreationally in their last sexual intercourse encounter/SDU—general type	(i) Lifetime and (ii) last use	Cross‐sectional; community; convenience; self‐administered questionnaire	16/41 (39%)	n.a	n.a	n.a
**32**	Boonchutima and Kongchan (2017) [70]	Thailand/upper‐middle income	9 February–10 March 2016	MSM dating apps users	Using substances during sexual intercourses/SDU—general type	Lifetime	Cross‐sectional; online; convenience; self‐administered questionnaire	39/350 (11.1%)	n.a	n.a	n.a Note: The study measured relationship using Pearson's correlation.
**33**	Schneiders and Weissman (2016)^a^ [71]	Cambodia/lower‐middle income	December 2012–January 2013	General MSM	Had sex, among those who used any drugs (Meth, amphetamine, heroin, inhalants, marijuana and ketamine) in the past 3 months/SDU—specific type	Past 3 months	Cross‐sectional; community; multi‐stage sampling; face‐to‐face interview	27/199 (13.57%)	n.a	n.a	n.a
**34**	Yeo and Ng (2016)^a^ [72]	Hong Kong/high income	November 2014 and February 2015	Young MSM	Drug use (including poppers) before or during anal sex/SDU—general type	Past 6 months	Cross‐sectional; community and online methods; convenience; self‐administered questionnaire	17/213 (7.9%)	n.s	n.a	n.a
**35**	Chen et al. (2015)^a^, ^c^ [73]	China/upper‐middle income	August–November 2009	General MSM	Use of group of substances with a connection to a club, dance scene and rave culture, such as poppers (volatile nitrate^h^), codeine phosphate, ketamine, ecstasy/MDMA, GHB, cocaine and methamphetamine at some time before or during sex in the past 6 months/SDU—specific type	Past 6 months	Cross‐sectional; community and online; venue‐based sampling, snowball, peer referral; self‐administered questionnaire	177/826 (21.4%)	n.s	1.93 (1.22–3.03)	Age <25 (aOR 3.6, 1.8–7.3, *p*<0.001); age 25–35 (aOR 3.2, 1.6–6.3, *p*<0.01); education middle school or less (aOR 3.7, 2.3–5.9, *p*<0.001); seeking sex partner through internet (aOR 1.8, 1.1–3.1, *p*<0.05); seeking sex partner through bars (aOR 4.8, 3.1–7.3, *p*<0.001); group sex 6PM (aOR 1.8, 1–3.3, *p*<0.05) , sex partners ≥ 2–4 (aOR 1.8, 1.3–2.5, *p*<0.001); sex partner >5 (aOR 2.6, 1.3–5.1, *p*<0.01); syphilis (OR 2.2, 1.3–3.8 *p*<0.005); STD‐related symptoms (OR 2.1, 1.4–3.3, *p*<0.001)
**36**	Yan et al. (2015)^a^, ^c^ [74]	China/upper‐middle income	April 2012–March 2013	General MSM	Ever having sex after using drugs during the past 6 months/SDU—general type	Past 6 months	Cross‐sectional; community; venue‐based sampling and online methods; face‐to‐face interview	37/306 (12.1%) general MSM^f^ 113/535 (21.2%) MSM sex workers	n.a	4.36 (1.72–11.07)	n.a
**37**	Lim et al. (2015)^a^ [22]	Malaysia/upper‐middle income	1 January 2010–28 February 2010	General MSM	Use of substances (including “poppers” [amyl nitrite^g^], ecstasy, crystal meth, marijuana, erectile dysfunction medications, cocaine, GHB [gamma hydroxybutyrate] and ketamine) prior to sex/SDU—specific type	Past 6 months	Cross‐sectional; online; convenience; self‐administered questionnaire	169/1235 (13.7%)	n.a (only aOR)	n.a (only aOR)	Sex partners >6 (aOR 4.8, 1.9–12.2, *p*<0.05); group sex (aOR 4.1, 2.3–7.1, *p*<0.05); any STIs (aOR 3.9, 1.7–9.1, *p*<0.05)
**38**	Huang et al. (2015) [76]	Taiwan/high income	2012	General MSM	Sexual contact after illegal drug use (inc. ketamine, MDMA, nimetazepam and others)/SDU—specific type	Lifetime	Cross‐sectional; community; convenience; self‐administered questionnaire	124/1208 (10.2%)	n.a	n.s	n.a
**39**	Cai and Lau (2014) [77]	Hong Kong/high income	March–October 2009	General MSM	The use of substances prior to having anal intercourse with the regular partner in the last 6 months/ SDU—general type	Past 6 months	Cross‐sectional; community; respondent‐driven sampling; face‐to‐face interview	35/211 (16.5%)	2.46 (1.15–5.26)	n.a	n.a
**40**	He et al. (2014) [78]	China/upper‐middle income	June and December of 2010	MSM HIV positive	Alkyl nitrite^i^ use and illicit drug use before having sex during the previous 6 months/ SDU—general type	Past 6 months	Cross‐sectional; community; snowball; face‐to‐face interview	39/200 (19.5%)	n.a	n.a	n.a
**41**	Li et al. (2014) [79]	China/upper‐middle income	July–October 2012	General MSM	Having sex after nitrate inhalants use/poppers—specific single drug type	Lifetime	Cross‐sectional; community and online methods; peer referral, web advertisement, community outreach, clinics; audio computer self‐interview	186/400 (46.7%)	n.s	2.88 (1.17–7.11)	Protected anal sex with casual partner in recent NI use (OR 0.5, 0.3–0.9, *p*<0.05); year of schooling >12 (OR 2.3, 1.3–4.1, *p*<0.01); internet for seeking sex partner (OR 3.5, 1.7–7.0, *p*<0.01; >1 male sex partner (OR 2.1, 1.3–3.6, *p*<0.01)
**42**	Van Griensven et al. (2013)^c^ [80]	Thailand/upper‐middle income	April 2006 and November 2010	General MSM	Drug use (including cannabis, MDMA, amphetamine, methamphetamine, PCP, cocaine, opiates and benzo diazepam) for sexual pleasure/SDU—specific type	Past 4 months	Prospective cohort; community and online methods; convenience; audio computer self‐interview	306/1744 (17.5%)	n.a	2.25 (1.71–2.95)	n.a
**43**	Koerner et al. (2012)^a^, ^c^ [81]	Japan/high income	November and December 2009	MSM gay bar customer	Use of any of a list of eight illicit and erectile maintenance drugs used during sex in the past 6 months/SDU—specific type	Past 6 months	Cross‐sectional; community; convenience; self‐administered questionnaire	156/723 (21.5%)	1.74 (1.19–2.54)^d^/past 6 months	n.a	n.a
**44**	Lim et al. (2012) [82]	Multi‐Asian countries (China, Singapore, Malaysia, Taiwan, Hong Kong, Thailand, Japan, Indonesia, the Philippines and other)/mix	1 January–28 February 2010	General MSM	Ever having used recreational drugs before sex in the past 6 months/SDU—general type	Past 6 months	Cross‐sectional; online; convenience; self‐administered questionnaire	1439/10,413 (13.8%)	n.a (only aOR)	n.a	n.a
**45**	Holtz et al. (2012) [83]	Thailand/upper‐middle income	April 2006–March 2010	General MSM	Ever use of methamphetamine to enhanced sex/meth Ever use of poppers to enhanced sex/poppers Ever used drugs (inc. meth, poppers and club drugs) to increase sexual pleasure/SDU—specific type. Ever use club drugs (cannabis, ecstasy [MDMA], amphetamine, methamphetamine, ketamine, cocaine and gamma‐hydroxybutyrate) to enhanced sex	Lifetime	Cross‐sectional, online and community method, convenience, audio computer self‐interview	52/1541 (3.4%) (meth) 214/1541 (13.8%) (poppers) 100/1541 (6.5%) (club drugs) 261/1541 (16.9%) (SDU)	n.a	n.a	*Treponema pallidum* positivity (OR 2.13, 1.24–3.65, *p*<0.05); HSV‐2 positive (OR 1.89, 1.12–2.94, *p*<0.05)
**46**	Wei et al. (2012) [84]	Multi‐Asian countries (Taiwan, Thailand, Singapore, Malaysia, China, Japan and Hong Kong)/mix	1 January and 28 February 2010	MSM HIV positive	Use of recreational drugs before sex in the past 6 months/SDU—general type	Past 6 months	Cross‐sectional; online; convenience; self‐administered questionnaire	152/401 (37.9%)	Infrequent SDU 2.20 (1.25–3.6); monthly of more SDU 7.24 (2.54–20.63)/past 6 months	n.a	n.a
**47**	Morineau et al. (2011) [85]	Indonesia/upper‐middle income	August and November 2007	General MSM	Use of methamphetamines or similar drugs before having sex in the 3 months prior the survey/meth—specific single drug type	Past 3 months	Cross‐sectional, community, venue random sampling and RDS, face‐to‐face interview	211/1450 (14.6%)	n.a	2.77 (1.38–5.56)	Consistent condom use (OR 0.54, 0.35–0.83, *p*<0.01)
**48**	Van Griensven et al. (2010)^a^ [86]	Thailand/upper‐middle income	April–May 2007	General MSM	Use of drugs during last sex/SDU—general type	Past 3 months	Cross‐sectional, community, venue‐day‐time sampling; self‐administered questionnaire	22/400 (5.5%)	n.a	n.a	n.a
**49**	Lau et al. (2009) [87]	Hong Kong/high income	Dec 2007–Feb 2008	MSM sex workers	Use of psychoactive substances before having sex with Hong Kong male clients/SDU—general type	Past 6 months	Cross‐sectional, community, convenience, face‐to‐face interview	45/199 (22.6%)	n.a	n.a	n.a (aOR 5.21 (2.29–11.84)/past 6 months)

Abbreviations: n.a, not assessed; n.s, not significant.

^a^ Included in meta‐analysis prevalence.

^b^ Included in meta‐analysis UAI.

^c^ Included in meta‐analysis HIV.

^d^ Manually calculated.

^e^ Used the SDU estimate in the past 12 months only.

^f^ Used the general MSM only for meta‐analysis.

^g^ Amyl nitrate: chemical compound with the formula C5H11ONO—part of the alkyl nitrate compound group—generally used to produce *poppers*.

^h^ Volatile nitrites: chemical compounds that evaporate contains nitrate (NO)—generally used to produce *poppers*.

^i^ Alkyl nitrate: group of chemical compounds based on molecular structure R‐ONO—generally used to produce *poppers*.

The majority of studies were cross‐sectional (*n* = 45) and the remainder (*n* = 4) were prospective cohort studies. Twenty‐five studies recruited participants from community settings, including MSM cruising sites or referral from civil society organizations, 10 recruited participants exclusively online (e.g. website banner advertising, online outreach, electronic mailers sent through gay community networks and location‐based social network mobile apps), 10 used a combination of community and online methods and four recruited from clinics. Most of the studies employed non‐probability sampling methods (*n* = 43), such as convenience, snowball and/or peer referral. Six studies employed probability methods, including respondent‐driven sampling [77, 85, 99], or venue‐time‐based sampling [56, 73, 74, 85, 86]. Data were collected through self‐administered surveys (*n* = 29), face‐to‐face researcher‐administered surveys (*n* = 14) or self‐completed computer/telephone‐assisted surveys (*n* = 6).

### 3.2 Pattern of SDU

SDU was inconsistently defined, with heterogeneity resulting from survey questions, varying descriptions of sexual context and drugs used. Twenty‐five studies provided checklists of specific drugs (e.g. *poppers*, ecstasy, crystal meth, marijuana, erectile dysfunction medications, cocaine, GHB and/or ketamine), with the selection of at least one drug used to classify SDU. Eight studies assessed single drug use, namely methamphetamine (*n* = 4) and poppers (*n* = 6). The remaining studies utilized general definitions that relied on participants’ self‐reports based on personal interpretations of what constitutes drug use (*n* = 24) that were then later categorized into groups: illegal drug use (*n* = 2); psychoactive drug (*n* = 2); and recreational drug (*n* = 2). The sexual contexts of SDU were defined as using drugs “before sex” (*n* = 14), “during sex” (*n* = 12), “for sex” (*n* = 10) or “before/during sex” (*n* = 13) (Table 1).

The most common recall period for SDU was past 6 months (*n* = 20). Other recall periods included lifetime (*n* = 14), past 3 months (*n* = 6), past 12 months (*n* = 5), past 4 months (*n* = 2), past month (*n* = 1) and last sex (*n* = 1). Duration of recall period for SDU impacted measured prevalence, with longer recall periods generally being associated with greater prevalence. Prevalence of SDU was generally greater when reported among specific groups of MSM (i.e. MSM living with diagnosed HIV, MSM aged 25 or younger and MSM sex workers) compared to general MSM (Table 1).

Seventeen studies measured sexual and other behavioural factors associated with SDU. Eight studies found associations between SDU with an increased number of male sex partners and sex with non‐regular partners (Table 1). SDU was also significantly associated with engaging in group sex (three studies) and having a history of STI diagnosis (six studies). Aside from CAI (see Section 3.4), other sexual risk behaviours were inconsistently defined and/or were measured only in the context of the sexualized use of poppers and, therefore, could not be included in the meta‐analysis. Additional factors associated with SDU included elevated use of alcohol before sex [55, 66], intimate partner violence [21, 57] and suicidal behaviour [52] (Table 1).

### 3.3 Prevalence of SDU

**Figure 2:**
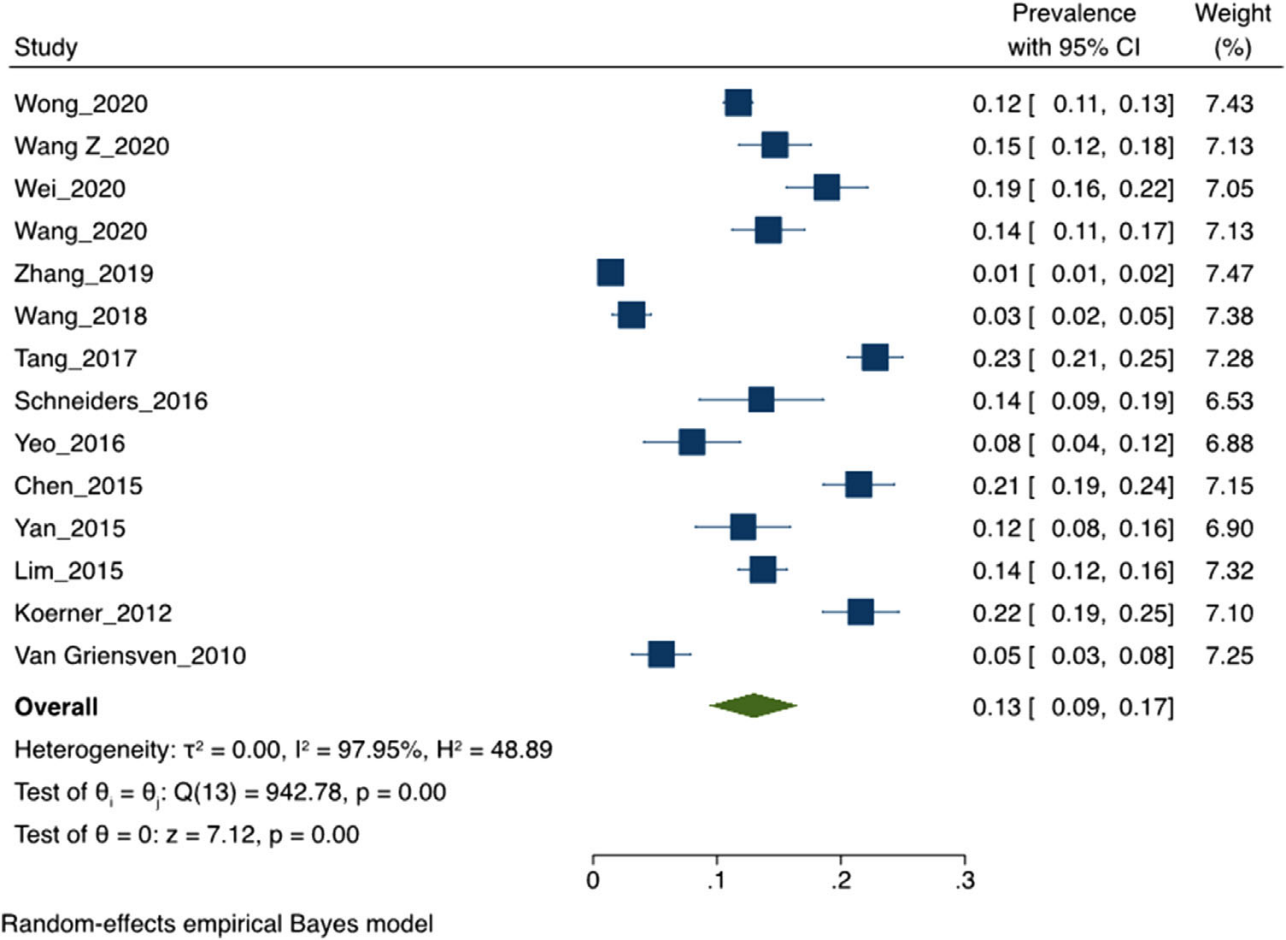
Forrest plot pooled sexualized drug use practice in the past 12 months using random effect empirical Bayes model. This presents a Forest plot identifying the basic components of the 14 studies included in the meta‐analysis of SDU prevalence. The square icon represents the individual study effect. The size of the square varies to reflect the weight a particular study has in the overall analysis (a larger square has more weight). The black line represents the CIs of a study; the smaller squares, which have less weight, generally have larger CIs than the square. The diamond represents the overall summary effect of SDU prevalence. The outer edges of the diamond represent the CIs.

Eighteen studies [16, 21, 74, 75, 55, 56, 61, 62, 64, 71–73] with 14,332 MSM participants were included in the meta‐analysis of recent SDU prevalence. The pooled prevalence of recent SDU among MSM in these studies was 13% (95% CI 10–16%) (Figure 2). High heterogeneity was observed (*I* ^2^ = 97.6%, *p*<0.001, *Q* = 1025.3). The test for subgroup differences revealed the estimated SDU prevalence was higher in self‐administered, or computer‐assisted data collection methods (15%; 95% CI 12–19%, *p*<0.05) compared to interviewer‐administered questionnaires (7%; 95% CI 1–13%; *p*<0.05) (Table 2). As expected, SDU prevalence increased in studies that specified the types of drugs utilized in the practice (15%) compared to those using general definitions (10%), although the difference here was not statistically significant (*p* = 0.08). Likewise, SDU prevalence increased as the assessed recall period increased: 9% for the past 3 months, 12% for the past 6 months and 17% for the past 12 months. However, these differences were not significant. Sensitivity analysis showed little effect on the overall pooled estimate when removing any one study from our initial model. There was no evidence of publication bias based on a visual assessment of the relative level of symmetry in the funnel plot and Begg's test for small‐study effects non‐parametric rank correlation (*p* = 0.039) (see Appendices S3 and S4).

**Table 2:** Stratified meta‐analysis of the proportion of recent SDU practice among MSM in East and South Asian countries

Subgroups/heterogeneity between groups	Prevalence % (95% CI)	# studies/sample	Heterogeneity within group *Q*; *I* ^2^ (%) ; *p*‐value
Pooled SDU prevalence	0.13 (0.10–0.16)	18/14,332	1025.3; 97.6 (< 0.001)
Data collection method (*Q* = 4.11; *p*= 0.043)*			
Interviewer administered questionnaire	0.07 (0.12–0.13)	4/2662	97.6% (< 0.001)
Self‐adm + comp. assisted	0.14 (0.11–0.17)	14/11,670	97.8% (< 0.001)
Drugs utilized in SDU (*Q* = 3.03; *p*= 0.08)			
Specific definition	0.15 (0.11–0.19)	9/9225	96.5% (< 0.001)
General definition	0.10 (0.6–0.14)	9/5107	97% (< 0.001)
Age group (*Q* = 0.34; *p* = 0.559)			
< 30 years old	0.14 (0.09–0.19)	7/4489	96% (< 0.001)
> 30 years old	0.12 (0.08–0.16)	11/9843	98% (< 0.001)
Recall period (*Q* =3.38; *p*=0.184)			
Past 3 months	0.09 (0.03–0.17)	2/599	87.8 (< 0.005)
Past 6 months	0.12 (0.08–0.16)	13/11,129	98.1 (< 0.001)
Past 12 months	0.17 (0.12–0.22)	3/2604	92.1 (< 0.001)
Economic level (*Q* = 0.43; *p* = 0.805)			
High income	0.14 (0.10–0.18)	5/5159	93.1% (< 0.001)
Upper middle	0.12 (0.08–0.16)	12/8974	98.2% (< 0.001)
Lower middle	0.14 (0.09–0.16)	1/199	–
Sampling method (*Q* = 1.03; *p*= 0.311)			
Probability	0.10 (0.05–0.15)	3/905	82.1% (< 0.005)
Non‐probability	0.13 (0.10–0.17)	15/13,427	98.1% (< 0.01)
Recruitment (*Q* = 0.29; *p* = 0.590)			
Offline	0.12 (0.07–0.17)	9/5461	97.8% (< 0.001)
Online/mix online and offline	0.14 (0.10–0.17)	9/8871	96.5% (< 0.001)
Geographical locations (*Q* = 2.92; *p*= 0.088)			
Single location	0.10 (0.06–0.15)	9/5509	98.2% (< 0.001)
Multilocations	0.15 (0.12–0.18)	9/8823	93.7% (< 0.001)
Study quality (*Q* = 1.55; *p*= 0.213)			
Lower quality	0.12 (0.08–0.16)	11/9550	98.2% (< 0.001)
Higher quality	0.14 (0.10–0.18)	7/4782	95.6% (< 0.001)

*p‐value <0.05.

### 3.4 SDU and CAI

**Figure 3:**
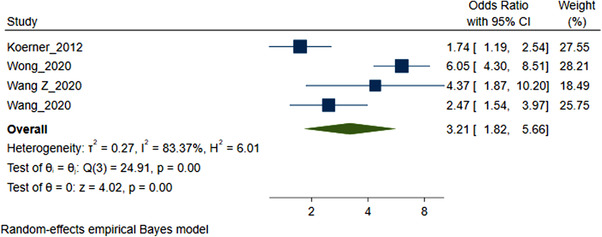
Forest plot pooled odds ratio sexualized drug use and condomless anal sex. This presents a Forest plot identifying the basic components of the four studies included in the meta‐analysis OR SDU and CAS. The square icon represents the individual study effect. The size of the square varies to reflect the weight a particular study has in the overall analysis (a larger square has more weight). The black line represents the CIs of a study; the smaller squares, which have less weight, generally have larger CIs than the square. The diamond represents the overall summary effect (OR CAS). The outer edges of the diamond represent the CIs.

Four studies (4946 MSM participants) included in the meta‐analysis of SDU prevalence reported a statistically significant association between SDU and CAI [16, 55, 56, 81]. The pooled OR for the association between CAI and SDU was 3.21 (95% CI 1.82–5.66) (Figure 3). Statistical heterogeneity across studies was high (*I* ^2^ 83.37%, *Q*(3) = 24.91, *p* <0.001).

### 3.5 SDU and HIV status

**Figure 4:**
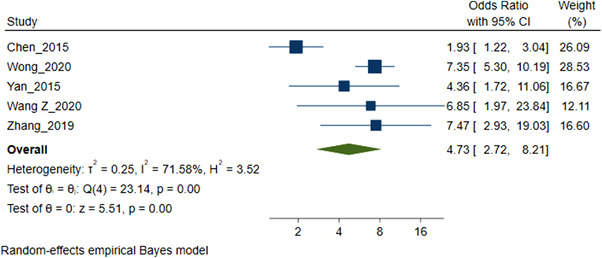
Forest plot pooled odds ratio sexualized drug use and HIV status. This depicts a Forest plot identifying the basic components of the five studies included in the meta‐analysis SDU and HIV status. The square icon represents the individual study effect. The size of the square varies to reflect the weight a particular study has in the overall analysis (a larger square has more weight). The black line represents the CIs of a study; the smaller squares, which have less weight, generally have larger CIs than the square. The diamond represents the overall summary effect (OR HIV status). The outer edges of the diamond represent the CIs.

Five studies (6386 MSM participants) included in the meta‐analysis of SDU prevalence reported a statistically significant association between SDU and HIV‐positive status [55, 56, 61, 73, 74]. The pooled OR for the association between being diagnosed with HIV and SDU was 4.73 (95% CI 2.72–8.21) (Figure 4). Statistical heterogeneity across studies was moderate (*I* ^2^ 71.58% *Q*(4) = 23.14, *p*<0.01).

### 3.6 Quality assessment

The level of bias across studies included in the meta‐analysis was classified as moderate. The rating of study bias was affected by convenience sampling being used in most studies, reliance on participant self‐reporting and limited information provided on participant response rates. A sensitivity analysis indicated that studies with lower quality tended to have a slightly higher SDU prevalence (see Table 2 and Appendix S5).

## 4 DISCUSSION

The findings of this review add to the existing literature by characterizing quantitative studies of SDU among MSM in East and South Asia and providing an estimate of SDU prevalence and its associations with sexual risk and HIV status in the region. The prevalence of SDU among MSM was comparable to that reported in other regions, albeit with variation in drug types used to define SDU, and the practice was associated with a range of sexual risk practices. In studies that were suitable for inclusion in meta‐analyses, an estimated 13% of MSM reported engaging in recent SDU, and SDU was associated with reporting CAI and living with diagnosed HIV. However, a lack of consistency in SDU measurement and definition is hampering the development of a coherent body of evidence surrounding SDU practice in East and South Asia.

This review identified that the prevalence of SDU was lower in the studies that utilized interview‐administered data collection methods. Drug use is regarded as a sensitive topic, with responses being affected by social desirability bias [100, 101]. The interview strategy, which entails social interaction with others, may compromise anonymity and confidentiality in terms of drug use history, therefore, affecting data quality. Furthermore, the prevalence was lower in shorter recall periods. While we cannot discount the influence of recall bias associated with longer recall periods, this finding is consistent with those from previous studies [102, 103]. This suggests that SDU for many MSM in East and South Asia (i.e. MSM who reported SDU across longer recall periods that would not otherwise have reported SDU across a short recall period) may be episodic or opportunistic. For those who engage purposively and frequently in SDU, the application of punitive approaches to drug‐related offences in most Asian countries influences the setting in which SDU is practiced [104, 105]. Reports of SDU taking place in secretive locations, concealed by coded language and promoted through online applications [106], suggest that the practice may be largely confined to relatively closed networks of MSM. While such insights help inform targeted health promotion and preventive harm reduction interventions, they also highlight the potential barriers to programme delivery. Criminalization and the clandestine nature of drug use have forced MSM who engage in SDU to remain “under the radar,” with programme engagement and disclosure of drug use practices impeded by fear and potential distrust of agencies delivering such programmes [107].

The implications of inconsistent quantitative measurements of SDU for determining the prevalence and correlates have been previously stated [108]. While the difference in SDU prevalence between studies that asked about specific drug types versus generalized questions of any drug use fell short of statistical significance, SDU prevalence in the former was, on average, 50% greater and there has been a call for a need to adopt consistent terminology related to SDU to better understand this practice [27, 109, 110]. The lack of clarity in defining the construct of SDU was also recognized in a qualitative review of SDU practice among MSM and transgender women in Asia [106]. Alongside consistent approaches to defining and measuring SDU, local drug markets and cultural contexts that shape SDU also need to be closely considered in research and practice. For example, it was common for studies in this review to include the use of a combination of drug types that included poppers within definitions of SDU. Poppers are not typically included in definitions of *chemsex* in Europe, despite being more commonly used by MSM in the context of sex than other forms of recreational drug use [111, 112]. There is evidence of an association between using poppers and higher‐risk sexual behaviours and elevated HIV risk [113, 114], and this supports their inclusion in polydrug SDU definitions in Asia. Historically, much of the literature related to SDU has emerged through the narrowly defined construct of *chemsex*, predominately in Europe and the United Kingdom, which relies on “the specific ‘highs’ associated with crystal methamphetamine, cathinone, and GHB/GBL that provide the desired pleasure and disinhibition” [115; pg 4].

Studies in East and South Asia described in this review employed a broad definition to describe SDU that relied on local understandings of the availability and use of drug types utilized by men in SDU practice, including those identified in qualitative research or stakeholder feedbacks [16, 59, 71, 84, 85]. This consideration of a broader array of substances, including non‐specific drug definitions, such as “recreational” or “illicit,” is also likely to be influenced by different motivational contexts related to drug policy and/or sex between men in Asia compared to Europe. Besides “heights of pleasure” that are said to motivate engagement in *chemsex*, MSM in Asia also report engaging in SDU to prolong sex, cope with social and cultural marginalization and traumatic experiences, enhance body image and because of the popularity and normalization of SDU [106, 116, 117]. Taking account of the inclusion of broader drug types, SDU practice among MSM in East and South Asia is common and the prevalence is comparable with other regions [8, 119]. However, the inclusion of a broader range of specific drug types within the SDU practice in East and South Asia influences how the practice should be perceived and risk interpreted and responded to.

There were only a small number of studies suitable to estimate pooled associations of SDU with CAI (*n* = 4) and HIV status (*n* = 5), with strong associations found for both. Our narrative review also identified common associations between SDU and other sexual risk practices, such as group sex or increasing number of sex partners. While these associations are often interpreted as evidencing a causative pathway between SDU, sexual risk practice and HIV acquisition, this implied temporality remains contestable due to the cross‐sectional study design. The association between SDU and reporting an HIV diagnosis may, in part, be due to initiating or increasing the frequency of SDU in response to testing positive for HIV, for example as a coping mechanism following HIV diagnosis [118], or due to changes in peer and sexual networks after HIV diagnosis [120] that may increase the exposure to SDU.

There are a number of limitations to this review. First, while there was no evidence of publication bias, limiting eligibility to English‐language publications in peer‐review databases may have excluded relevant articles published in non‐English language journals or in country‐level reports, especially when the review is focused on Asian regions where their first language is often not English. Second, 50% of the studies included in the SDU prevalence are from China, which may not reflect the broader behavioural and cultural contexts of MSM and drug use in other Asian countries. Yet, studies included in the review still had high heterogeneity. Also, only one of the 18 studies included lower‐middle economic countries biasing the results to more upper‐middle and high‐income countries. Third, because of differences in SDU definition, recall period, measurement of risk practices and disaggregation of findings, we were unable to include more studies in the meta‐analyses, which limited our ability to assess publication bias in CAI and HIV‐positive status meta‐analyses. Fourth, the majority of included studies did not specify the gender identity of their MSM participants. Therefore, we cannot confirm gender identity categories, that is cisgender MSM, transgender people or gender non‐conforming of MSM populations, in our review. Fifth, the majority of studies included are cross‐sectional, limiting our ability to assess causality between SDU and sexual risk and HIV positivity. Sixth, this review was not able to measure the pooled prevalence of single drug use (i.e. poppers and methamphetamine) in SDU practice due to a limited study sample. Lastly, all studies included in our review adopted different methodologies for study designs, data collection methods and sampling techniques, which may have contributed to the high heterogeneity among our study findings.

## 5 CONCLUSIONS

Our findings suggest that SDU is commonly practiced by some MSM in East and South Asian countries and is associated with sexual risk, including CAI and HIV seropositivity. The findings of this systematic review, therefore, support recommendations for tailored interventions to address the nexus between drug use and sexual risk among MSM in Asia, including the development of localized harm reduction messages targeting MSM who practice SDU and those who are at risk of engaging in SDU in the inclusion of SDU risk assessment as part of MSM outreach and STI and HIV services, in combination with promotion and provision of condoms, lubricants and PrEP as preventive methods. However, a lack of consistency in measuring SDU and associated outcomes makes cross‐study and between‐country comparisons challenging and this limits the development of generalized and tailored local responses. Situational and qualitative assessments of local SDU environments and norms are needed to understand the nature, context and implications of the practice and inform potential programmes for harm reduction [121]. This would also support the development of standardized approaches to measuring SDU that take account of local contexts, strengthen results comparability between studies and locations, and offer insights into how SDU can be sensitively measured and representative samples recruited in the context of perceived stigma within local communities. For community‐based organizations in Asia, practical and contextual guidance for responding to SDU practice is now available to guide the intervention [121]. The guideline marks a positive way forward to implement contextually specific SDU interventions.

## COMPETING INTERESTS

MS and AP have received investigator‐initiated research funding from Gilead Sciences and AbbVie and consultant fees from Gilead Sciences for activities unrelated to this work.

## AUTHORS’ CONTRIBUTIONS

All authors contributed to the study conception and design. Material preparation and data collection were performed by LN and SES. Analysis was performed by LN. The first draft of the manuscript was written by LN and all authors commented on previous versions of the manuscript. All authors read and approved the final manuscript.

## FUNDING

No funding was received to assist with the preparation of this manuscript. LN received scholarships for her doctoral degree from Indonesian Endowment Fund for Education (LPDP scholarship), Ministry of Finance, Republic of Indonesia. SS is the grateful recipient of an Australian Government Research Training Program (RTP) Scholarship and a Monash International Tuition Scholarship (MITS). MS is supported by a National Health and Medical Research Council Senior Research Fellowship.

## Supporting information


**Supplementary 1**: Database Search Syntax.
**Supplementary 2**: Quality appraisal scores of included observational studies according to Joanna Briggs Institute Critical Appraisal tools checklist for prevalence studies.
**Supplementary 3**: Sensitivity analysis using leave‐one‐out method for assessing the effect of a single study on SDU pooled prevalence result.
**Supplementary 4**: Publication bias assessment.
**Supplementary 5**: Sensitivity analysis by adding one study at a time to each subsequent analysis from lowest to highest quality studies.

## Data Availability

The data that support the findings of this study are available from the corresponding author upon reasonable request.
