# Supplementary material for: Prevalence of sexualized drug use and risk of HIV among sexually active MSM in East and South Asian countries: systematic review and meta‐analysis
Source: J Int AIDS Soc. 2023 Jan 4;26(1):e26054. doi: 10.1002/jia2.26054 (PMC9813405; doi:10.1002/jia2.26054)

Appendices

**PREVALENCE OF SEXUALISED SUBSTANCE USE AND RISK OF HIV AMONG SEXUALLY ACTIVE MSM IN EAST AND SOUTH ASIAN COUNTRIES: SYSTEMATIC REVIEW AND META-ANALYSIS**

Table of Contents

[Supplementary 1: Database Search Syntax 2](#_Toc98249625)

[Supplementary 2. Quality appraisal scores of included observational studies according to Joanna Briggs Institute Critical Appraisal tools checklist for prevalence studies 7](#_Toc98249626)

[Supplementary 3. Sensitivity analysis using leave-one-out method for assessing the effect of a single study on SDU pooled prevalence result. 8](#_Toc98249627)

[Supplementary 4. Publication bias assessment 9](#_Toc98249628)

[Supplementary 5: Sensitivity analysis by adding one study at a time to each subsequent analysis from lowest to highest quality studies. 10](#_Toc98249629)

**Supplementary 1: Database Search Syntax**

**The searches were conducted in August 2020 and updated on September 26^th^, 2022**

**OVID Medline (703 results)**

**OVID Embase (1155 results)**

**OVID Global Health (694 results)**

**Psyinfo (213 results)**

| **1** | (MSM or men who have sex with men or gay or gender minorit* or male homosexual* or men having sex with men or homosexuality or sexual minorit* or bisexual* or glb* person* or gays or lbg* person* or non-heterosexual* or queer male* or queer men or males who have sex with males or males having sex with males).mp. |
| --- | --- |
| **2** | (chemsex or chem-sex or chem-fun or sexuali#ed substance* or sexuali#ed drug* or party-n-play or "party and play" or PNP or "high fun" or "recreational drug*" or "drug* use" or "substance* use" or "intravenous drug*").mp. |
| **3** | (methamphetamine* or amphetamine* or puff or cocaine or illicit drug* or psychotropic drug* or psychoactive substance* or "central nervous system stimulant*" or mephedrone or ghb or gamma-hydroxybutyric acid or gamma hydroxybutyrate or poppers or alkyl nitrites or rush or crystal-meth or mdma or ecstasy or cocaine, cannabis or heroin or opioid* or morphine or erectile dysfunction drug* or viagra or blue diamond or new psychoactive substance* or party drug* or new psychoactive drug* or performance-enhancing drug* or performance-enhancing substance*).mp. |
| **4** | (prep or pre-exposure profilax* or pep or post-exposure profilax* or pre-exposure prophylax* or post-exposure prophylax*).mp. |
| **5** | **1 and 2** |
| **6** | **1 and 3** |
| **7** | **1 and 4** |
| **8** | (asia or south-east asia or south asia or japan or china or indonesia or india or south korea or thailand or vietnam or singapore or philippines or hong kong or malaysia or pakistan or mongolia or taiwan or myanmar or cambodia or nepal or bangladesh or afghanistan or maldives or sri lanka or north korea or laos or timor-leste or brunei or bhutan or chinese or japanese or thai).mp. |
| **9** | (5 or 6 or 7) and 8 |
| **10** | limit 9 to (english language and yr="1990 -Current") |

**CINAHL (274 results)**

| **S1** | TX MSM or men who have sex with men or gay or gender minorit* or male homosexual* or men having sex with men or homosexuality or sexual minorit* or bisexual* or glb* person* or gays or lbg* person* or non-heterosexual* or queer male* or queer men or males who have sex with males or males having sex with males | Search modes - Boolean/Phrase |
| --- | --- | --- |
| **S2** | TX chemsex or chem-sex or chem-fun or sexuali#ed substance* or sexuali#ed drug* or party-n-play or “party and play” or PNP or “high fun” or “recreational drug*” or drug* use or substance* use or intravenous drug* | Search modes - Boolean/Phrase |
| **S3** | TX methamphetamine* or amphetamine* or puff or cocaine or illicit drug* or psychotropic drug* or psychoactive substance* or “central nervous system stimulant*” or mephedrone or ghb or gamma-hydroxybutyric acid or gamma hydroxybutyrate or poppers or alkyl nitrites or rush or crystal-meth or mdma or ecstasy or cocaine, cannabis or heroin or opioid* or morphine or erectile dysfunction drug* or viagra or blue diamond or new psychoactive substance* or party drug* or new psychoactive drug* or performa | Search modes - Boolean/Phrase |
| **S4** | TX prep or pre-exposure profilax* or pep or post exposure profilax* or pre-exposure prophylax* or post exposure prophylax* | Search modes - Boolean/Phrase |
| **S5** | S1 AND S2 | Search modes - Boolean/Phrase |
| **S6** | S1 AND S3 | Search modes - Boolean/Phrase |
| **S7** | S1 AND S3 | Search modes - Boolean/Phrase |
| **S8** | AB asia or south-east asia or south asia or japan or china or indonesia or india or south korea or thailand or vietnam or singapore or philippines or hong kong or malaysia or pakistan or mongolia or taiwan or myanmar or cambodia or nepal or bangladesh or afghanistan or maldives or sri lanka or north korea or laos or timor-leste or brunei or bhutan or chinese or japanese or thai | Search modes - Boolean/Phrase |
| **S9** | S5 OR S6 OR S7 | Search modes - Boolean/Phrase |
| **S10** | S8 AND S9 | Search modes -Boolean/Phrase |
| **S11** | S8 AND S9 | Narrow by Language: - English - Search modes - Boolean/Phrase |

**SCOPUS (323 results)**

| **1** | **ALL ( msm OR "men who have sex with men" OR gay OR "gender minorit*" OR "male homosexual*" OR "men having sex with men" OR homosexuality OR "sexual minorit*" OR bisexual* OR glb* AND person* OR gays OR lbg* AND person* OR "non-heterosexual*" OR "queer male*" OR "queer men" OR "males who have sex with males" OR "males having sex with males" )** |
| --- | --- |
| **2** | **ALL ( chemsex OR chem-sex OR chem-fun OR "sexuali#ed substance*" OR "sexuali#ed drug*" OR party-n-play OR "party and play" OR pnp OR "high fun" OR "recreational drug*" OR "intravenous drug*" )** |
| **3** | **ALL ( methamphetamine* OR amphetamine* OR puff OR cocaine OR "illicit drug*" OR "psychotropic drug*" OR "psychoactive substance*" OR "central nervous system stimulant*" OR mephedrone OR ghb OR gamma-hydroxybutyric AND acid OR gamma AND hydroxybutyrate OR poppers OR "alkyl nitrites" OR rush OR crystal-meth OR mdma OR ecstasy OR cocaine OR cannabis OR heroin OR opioid OR morphine OR "erectile dysfunction drug*" OR viagra OR "blue diamond" OR "new psychoactive substance*" OR "party drug*" OR "new psychoactive drug*" OR "performance enhancing drug*" OR "performance enhancing substance*" )** |
| **4** | **ALL ( prep OR "pre-exposure profilax*" OR pep OR "post exposure profilax*" OR "pre-exposure prophylax*" OR "post exposure prophylax*" )** |
| **5** | **( ALL ( msm OR "men who have sex with men" OR gay OR "gender minorit*" OR "male homosexual*" OR "men having sex with men" OR homosexuality OR "sexual minorit*" OR bisexual* OR glb* AND person* OR gays OR lbg* AND person* OR "non-heterosexual*" OR "queer male*" OR "queer men" OR "males who have sex with males" OR "males having sex with males" ) ) AND ( ALL ( chemsex OR chem-sex OR chem-fun OR "sexuali#ed substance*" OR "sexuali#ed drug*" OR party-n-play OR "party and play" OR pnp OR "high fun" OR "recreational drug*" OR "intravenous drug*" ) )** |
| **6** | **( ALL ( msm OR "men who have sex with men" OR gay OR "gender minorit*" OR "male homosexual*" OR "men having sex with men" OR homosexuality OR "sexual minorit*" OR bisexual* OR glb* AND person* OR gays OR lbg* AND person* OR "non-heterosexual*" OR "queer male*" OR "queer men" OR "males who have sex with males" OR "males having sex with males" ) ) AND ( ALL ( methamphetamine* OR amphetamine* OR puff OR cocaine OR "illicit drug*" OR "psychotropic drug*" OR "psychoactive substance*" OR "central nervous system stimulant*" OR mephedrone OR ghb OR gamma-hydroxybutyric AND acid OR gamma AND hydroxybutyrate OR poppers OR "alkyl nitrites" OR rush OR crystal-meth OR mdma OR ecstasy OR cocaine OR cannabis OR heroin OR opioid OR morphine OR "erectile dysfunction drug*" OR viagra OR "blue diamond" OR "new psychoactive substance*" OR "party drug*" OR "new psychoactive drug*" OR "performance enhancing drug*" OR "performance enhancing substance*" ) )** |
| **7** | **( ALL ( msm OR "men who have sex with men" OR gay OR "gender minorit*" OR "male homosexual*" OR "men having sex with men" OR homosexuality OR "sexual minorit*" OR bisexual* OR glb* AND person* OR gays OR lbg* AND person* OR "non-heterosexual*" OR "queer male*" OR "queer men" OR "males who have sex with males" OR "males having sex with males" ) ) AND ( ALL ( prep OR "pre-exposure profilax*" OR pep OR "post exposure profilax*" OR "pre-exposure prophylax*" OR "post exposure prophylax*" ) )** |
| **8** | **TITLE-ABS-KEY ( asia OR south-east asia OR south asia OR japan OR china OR indonesia OR india OR south korea OR thailand OR vietnam OR singapore OR philippines OR hong kong OR malaysia OR pakistan OR mongolia OR taiwan OR myanmar OR cambodia OR nepal OR bangladesh OR afghanistan OR maldives OR sri lanka OR north korea OR laos OR timor-leste OR brunei OR bhutan OR chinese OR japanese OR thai)** |
| **9** | **((ALL( msm OR "men who have sex with men" OR gay OR "gender minorit*" OR "male homosexual*" OR "men having sex with men" OR homosexuality OR "sexual minorit*" OR bisexual* OR glb* AND person* OR gays OR lbg* AND person* OR "non-heterosexual*" OR "queer male*" OR "queer men" OR "males who have sex with males" OR "males having sex with males" ) ) AND ( ALL ( chemsex OR chem-sex OR chem-fun OR "sexuali#ed substance*" OR "sexuali#ed drug*" OR party-n-play OR "party and play" OR pnp OR "high fun" OR "recreational drug*" OR "intravenous drug*" ) ) ) OR ( ( ALL ( msm OR "men who have sex with men" OR gay OR "gender minorit*" OR "male homosexual*" OR "men having sex with men" OR homosexuality OR "sexual minorit*" OR bisexual* OR glb* AND person* OR gays OR lbg* AND person* OR "non-heterosexual*" OR "queer male*" OR "queer men" OR "males who have sex with males" OR "males having sex with males" ) ) AND ( ALL ( methamphetamine* OR amphetamine* OR puff OR cocaine OR "illicit drug*" OR "psychotropic drug*" OR "psychoactive substance*" OR "central nervous system stimulant*" OR mephedrone OR ghb OR gamma-hydroxybutyric AND acid OR gamma AND hydroxybutyrate OR poppers OR "alkyl nitrites" OR rush OR crystal-meth OR mdma OR ecstasy OR cocaine OR cannabis OR heroin OR opioid OR morphine OR "erectile dysfunction drug*" OR viagra OR "blue diamond" OR "new psychoactive substance*" OR "party drug*" OR "new psychoactive drug*" OR "performance enhancing drug*" OR "performance enhancing substance*" ) ) ) OR ( ( ALL ( msm OR "men who have sex with men" OR gay OR "gender minorit*" OR "male homosexual*" OR "men having sex with men" OR homosexuality OR "sexual minorit*" OR bisexual* OR glb* AND person* OR gays OR lbg* AND person* OR "non-heterosexual*" OR "queer male*" OR "queer men" OR "males who have sex with males" OR "males having sex with males" ) ) AND ( ALL ( prep OR "pre-exposure profilax*" OR pep OR "post exposure profilax*" OR "pre-exposure prophylax*" OR "post exposure prophylax*" ) ) ) AND ( TITLE-ABS-KEY ( asia OR south-east asia OR south asia OR japan OR china OR indonesia OR india OR south korea OR thailand OR vietnam OR singapore OR philippines OR hong kong OR malaysia OR pakistan OR mongolia OR taiwan OR myanmar OR cambodia OR nepal OR bangladesh OR afghanistan OR maldives OR sri lanka OR north korea OR laos OR timor-leste OR brunei OR bhutan OR chinese OR japanese OR thai ) )** |

## Supplementary 2. Quality appraisal scores of included observational studies according to Joanna Briggs Institute Critical Appraisal tools checklist for prevalence studies

| Study | *Risk of Bias Domain/Combined Score* | | | | | | | | |  | |
| --- | --- | --- | --- | --- | --- | --- | --- | --- | --- | --- | --- |
|  | **1** | **2** | **3** | **4** | **5** | **6** | **7** | **8** | **9** | **Total Score – Max. 100** |  |
| Wang, 2018 | 11 | 0 | 11 | 11 | 11 | 6 | 11 | 11 | 0 | **72** |  |
| Schneiders, 2020 | 11 | 0 | 0 | 6 | 11 | 6 | 11 | 6 | 0 | **50** |  |
| Tang, 2017 | 6 | 0 | 11 | 6 | 11 | 6 | 11 | 11 | 0 | **61** |  |
| Koerner, 2021 | 11 | 0 | 11 | 6 | 11 | 6 | 11 | 11 | 6 | **72** |  |
| Yeo, 2016 | 11 | 0 | 6 | 11 | 11 | 6 | 11 | 11 | 0 | **67** |  |
| Chen, 2015 | 11 | 0 | 6 | 11 | 11 | 6 | 11 | 11 | 0 | **67** |  |
| Wong, 2020 | 11 | 0 | 6 | 11 | 11 | 6 | 11 | 11 | 0 | **67** |  |
| Yan, 2015 | 11 | 0 | 6 | 11 | 6 | 6 | 11 | 11 | 0 | **61** |  |
| Wang, 2020 | 11 | 0 | 6 | 11 | 11 | 6 | 11 | 11 | 6 | **72** |  |
| Lim, 2015 | 11 | 0 | 6 | 11 | 11 | 6 | 11 | 11 | 6 | **72** |  |
| Wei, 2020 | 11 | 0 | 11 | 11 | 11 | 0 | 11 | 11 | 0 | **67** |  |
| Van Grivnsven, 2010 | 11 | 11 | 11 | 11 | 11 | 0 | 0 | 11 | 0 | **67** |  |
| Wang, 2020 | 6 | 6 | 11 | 11 | 11 | 6 | 6 | 11 | 6 | **72** |  |
| Zhang, 2019 | 11 | 0 | 11 | 6 | 11 | 0 | 11 | 11 | 0 | **61** |  |
| Zhang, 2022 | 11 | 6 | 6 | 11 | 6 | 0 | 11 | 11 | 6 | **67** |  |
| Eger, 2022 | 11 | 6 | 6 | 11 | 6 | 0 | 11 | 11 | 0 | **61** |  |
| Yang, 2022 | 11 | 11 | 11 | 11 | 6 | 6 | 11 | 11 | 11 | **89** |  |
| Duan, 2021 | 11 | 11 | 11 | 11 | 6 | 6 | 11 | 11 | 11 | **89** |  |
| Lee, 2021 | 11 | 11 | 6 | 11 | 6 | 6 | 11 | 11 | 6 | **78** |  |

##

## Supplementary 3. Sensitivity analysis using leave-one-out method for assessing the effect of a single study on SDU pooled prevalence result.


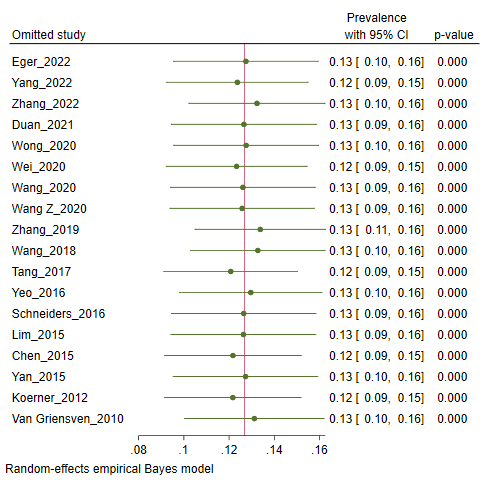


## Supplementary 4. Publication bias assessment


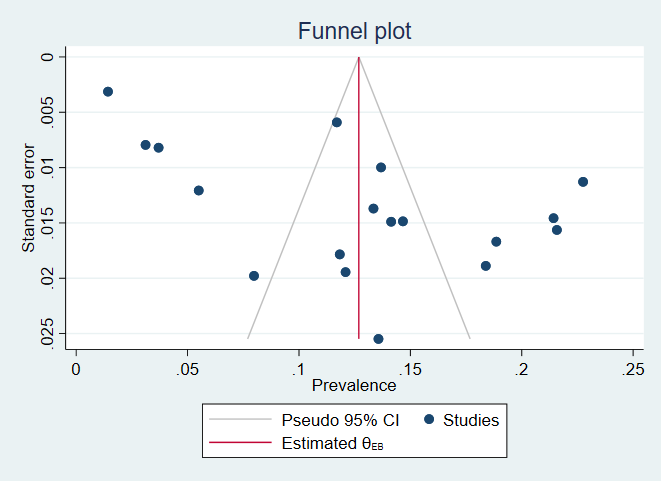


## Supplementary 5: Sensitivity analysis by adding one study at a time to each subsequent analysis from lowest to highest quality studies.


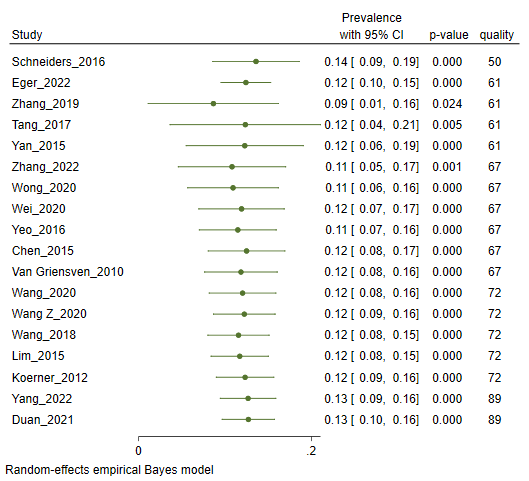

Supplement: Supplementary file 1 — Supplementary 1: Database Search Syntax. Supplementary 2: Quality appraisal scores of included observational studies according to Joanna Briggs Institute Critical Appraisal tools checklist for prevalence studies. Supplementary 3: Sensitivity analysis using leave‐one‐out method for assessing the effect of a single study on SDU pooled prevalence result. Supplementary 4: Publication bias assessment. Supplementary 5: Sensitivity analysis by adding one study at a time to each subsequent analysis from lowest to highest quality studies. [file JIA2-26-e26054-s001.docx]
